# A 6-month trial of memantine for nystagmus and associated phenomena in oculopalatal tremor

**DOI:** 10.3389/fneur.2022.921341

**Published:** 2022-08-17

**Authors:** Ana Inês Martins, Ricardo Soares-dos-Reis, André Jorge, Cristina Duque, Daniela Jardim Pereira, Carlos Fontes Ribeiro, João Sargento-Freitas, Anabela Matos, Luís Negrão, João Lemos

**Affiliations:** ^1^Department of Neurology, Coimbra University and Hospital Centre, Coimbra, Portugal; ^2^Department of Neurology, Centro Hospitalar Universitário de São João, Porto, Portugal; ^3^Department of Clinical Neurosciences and Mental Health, Faculty of Medicine, University of Porto, Porto, Portugal; ^4^Department of Neurology, Pedro Hispano Hospital, Matosinhos, Portugal; ^5^Department of Neuroradiology Unit/Imaging, Coimbra University and Hospital Centre, Coimbra, Portugal; ^6^Faculty of Medicine, Coimbra University, Coimbra, Portugal

**Keywords:** memantine, nystagmus, palatal tremor, oculopalatal tremor, Guillain-Mollaret triangle

## Abstract

**Introduction:**

Oculopalatal tremor (OPT) is a late manifestation of a Guillain-Mollaret triangle lesion. Memantine has been shown to improve nystagmus in OPT, but its long-term efficacy and putative distinct effects on each plane of nystagmus and on associated phenomena (e.g., gravity perception) are largely unknown.

**Methods:**

We conducted a 6-month open-label study to evaluate the effect of memantine in OPT patients. Baseline (visit 1), 2 (visit 2), and 6 months (visit 3) assessments included video-oculography, best corrected visual acuity (BCVA), visual function questionnaire (VFQ25), palatal tremor frequency, and subjective visual vertical (SVV). Memantine was titrated to 20 mg per day and stopped after 6 months.

**Results:**

We included six patients (5 females; mean age 68.5+/-9.7). At visit 2, nystagmus improved >50% only along the horizontal plane in two patients, while worsening >50% along the vertical and horizontal planes in 4 and 1 patients, respectively. At visit 3, previous improvement of nystagmus along the horizontal plane in two patients was not sustained, and it further worsened >50% along the vertical plane in 4. The mean vertical velocity and amplitude of nystagmus in the left eye significantly worsened from visit 2 to visit 3 (*p* = 0.028). Throughout the study, nystagmus frequency remained unchanged (*p* = 0.074), BCVA improved in both eyes (*p* = 0.047, *p* = 0.017), SVV progression was unpredictable (*p* = 0.513), and the mean VFQ-25 score (*p* = 0.223) and mean palatal frequency remained unchanged.

**Conclusion:**

The long-term use of memantine 20 mg per day in OPT produced a modest and only transient improvement in nystagmus, predominantly along the horizontal plane. Visual acuity improved, albeit without relevant changes in vision-related quality of life.

## Introduction

Oculopalatal tremor (OPT) is a rare and delayed complication of damage to the dentatorubro-olivary pathway—the Guillain and Mollaret's triangle (GMT). It is characterized by the presence of pendular nystagmus (PN), frequently synchronous with palate tremor (PT). Most patients exhibit predominantly vertical PN, accompanied by a variable degree of a horizontal and torsional nystagmus. PN is usually large, fast, irregular, and disconjugate between eyes (1). Jerk nystagmus might accompany PN, possibly due to damage to nearby structures (2). Importantly, patients with OPT-related PN complain of disturbing oscillopsia and decreased visual acuity, with deterioration of vision-specific health-related quality of life (1). PN in OPT has been attributed to an abnormal neuronal firing of the inferior olivary nucleus (ION), further amplified by the cerebellum, due to maladaptive cerebellar learning (2). Hypertrophy and T2 hyperintensity of the ION reflecting trans-synaptic degeneration is classically seen on MRI (3). Moreover, OPT patients also seem to show a deficit in estimating the direction of gravity (i.e., subjective visual vertical, SVV), possibly due to associated dentate nucleus (DN) dysfunction (4).

There is anecdotal data showing that memantine, an N-methyl-D-aspartate (NMDA) receptor antagonist, might reduce PN amplitude, velocity and frequency variability, and improve visual acuity and distance oscillopsia in some but not all patients with OPT-related PN (5–7). An additional increase in PN waveform randomness (i.e., waveform heterogeneity and visible differences in their shapes) may account for the idiosyncratic subjective visual response, often not correlating with an objective reduction of PN (6, 8). Jerk nystagmus has also been shown to improve with memantine (6). Importantly, all the above studies were small (4–6 OPT patients), with a short follow-up (15–21 days), frequently using memantine 40 mg per day, and usually only providing data from the dominant plane of PN (i.e., the plane [horizontal, vertical, or torsional] with the more regular and/or the largest amplitude of eye oscillation in each eye) as an outcome for therapeutic response (5–8). Almost all patients in the above series had at least one side effect while taking memantine 40 mg per day, including lethargy, drowsiness, maniac episode/confusional state, increased emotionality, irritability and anxiety, ataxia, and neuropathic pain (5–8). Therefore, lower doses have been recommended (5).

Given the previously reported disparity between amplitude reduction of the dominant plane of PN and the relatively mild or unpredictable subjective visual improvement in OPT patients, in this work, we asked if there are distinctive effects of memantine on horizontal and vertical components of nystagmus (including PN, and associated jerk nystagmus, if present) in individual patients, which could also help to explain the idiosyncratic subjective visual response seen in OPT (8). In addition, as data are sparse regarding long-term outcomes for memantine response in OPT, we evaluated the effect of memantine 20 mg per day in OPT patients during a 6-month period, focusing on several aspects of the outcome, including nystagmus intensity in each plane, visual acuity, SVV, PT and facial movements frequency, and quality of life measurements.

## Methods

### Study design and setting, protocol approvals, and patient consents

We conducted a single-center, open-label trial to test the effects of memantine in patients with OPT. The research followed the tenets of the Declaration of Helsinki. The study was approved by our local medical ethical committee, study number 41.2016. All patients were informed about the design and the purpose of the study. Patients provided informed, written consent to the protocol and study procedures.

### Participants

We included patients diagnosed with symptomatic OPT at our tertiary referral center. Patients with other ophthalmological disorders that could impair vision, unable to perform video-oculographic assessment, or those with ongoing seizures, severe neurological disability, psychiatric disorder, or other contraindication to memantine therapy, were excluded. Memantine was titrated through a 21-day period to 20 mg/day and stopped after 6 months. Patients under specific treatment for OPT before the study had to suspend it for at least 6 months before entering the study.

Patients were evaluated at baseline (visit 1, prior to memantine introduction), 2 months (visit 2), and 6 months (visit 3) post-treatment initiation. In each visit, patients underwent a complete neurologic, neuro-ophthalmologic and neuro-otologic exam (only visit 1), video-oculography (VOG), best corrected near visual acuity (BCVA) by using a Rosenbaum Eye Chart scale and then converted into a decimal scale, subjective visual vertical (SVV) assessment, visual function questionnaire (NEI-VFQ-25), and PT and facial movements assessment using video recordings and orbicularis oris/oculis and frontalis muscles electromyography (EMG). In visits 2 and 3, the assessment was performed at least 2 h after drug dosing. Side effects and subjective improvement of oscillopsia were ascertained by direct questioning of the participants. All patients underwent head MRI at the beginning of the study.

### Video-oculography

Eye movements were recorded using binocular video-oculography (Interacoustics VO425, Assen, Denmark; 105 Hz). After 5-point calibration, spontaneous nystagmus was assessed while fixating a 1.5-m distance centered target, for 30 s. The patient's head was manually restrained throughout the eye movement recording.

### Subjective visual vertical

SVV was assessed through the “bucket test”. The examiner rotated a bucket placed near the subject's head, clockwise and anticlockwise for six trials, asking the subject to re-rotate it until he/she perceived that the radiant line displayed inside the bucket was vertical. The angle of deviation was measured by an oscillometer attached on the outside. A mean of the result of six trials was taken as an absolute value (9).

### Vision-specific quality of life questionnaire

To evaluate the impact of nystagmus on quality of life, we used the 25-Item National Eye Institute Visual Functioning Questionnaire (NEI-VFQ-25) (10).

### Palatal tremor and facial movements

A 30-s videotape of the palatal movements was obtained during each visit. Additionally, when facial muscles other than soft palate/pharynx were also involved, these were electrographically recorded through EMG for 30 s (Nicolet Viking Quest 21.1, Middletown, USA).

### MRI

All patients were scanned using a 3T Magnetom Trio scanner (Siemens, Erlangen, Germany). The imaging protocol included at least: (1) high-resolution 3D T1 MPRAGE anatomical sequence; (2) 3 mm-thick axial FLAIR and DP/T2 for evaluation of IO hypertrophy and damage in other components of GMT; and (3) susceptibility-weighted imaging or T2^*^ gradient echo (GRE), since all the patients included in this study had brainstem or cerebellar hemorrhage.

### Analysis

VOG horizontal and vertical eye tracings from each eye were analyzed offline with custom software *(http://faculty.washington.edu/jokelly/voganalysis)*. After manual artifact rejection (i.e., blinks, drop-outs, saccades, and erroneous gaze shifts), mean amplitude, velocity, and frequency of nystagmus were computed in a stable baseline tracing (11, 12). The mean velocity was calculated using desaccaded tracings with an array size >2,000 points. The mean amplitude and frequency of nystagmus were calculated manually, by inspecting each cycle of nystagmus. In patients with a jerk form of nystagmus superimposed on the pendular form, amplitude and frequency were calculated separately for each form. As there were no differences when using either form, in the final results, only nystagmus amplitude and frequency data concerning pendular nystagmus are shown. PT frequency was calculated manually from each video. Facial movement frequency was analyzed with EMG built-in software.

For individual analysis of nystagmus, a significant change was defined as a 50% decrease (i.e., improvement) or increase (i.e., worsening) of velocity and/or amplitude between visits (5). For group analysis of nystagmus and other variables, related-samples Friedman test with *x*^2^ test statistics was run to determine whether there were differences in measured scores between baseline, 2 and 6-month visits. *Post hoc* analysis was conducted using the Wilcoxon signed-rank test. Spearman rank correlation coefficient was used to assess the relationships between tested variables at each visit. Statistical analysis was performed using SPSS version 22.0.0 (IBM, Armonk, NY). Differences were considered significant at p < 0.05.

## Results

### Demographic and clinical data

Six OPT patients reporting oscillopsia were included, mean age 68.5 ± 9.7, range 59–83 years, five females. Two additional patients declined to participate in the study. OPT's causative lesions were either pontine (5) or cerebellar (1) hemorrhages, which occurred with a mean time of 51.5 ± 15.4 months before study entry. All patients had additional ocular motor and/or neurological signs, apart from OPT. PT propagated to facial musculature in 3 patients. Nystagmus was mostly pendular vertical and torsional, and asymmetric between eyes. Associated jerk nystagmus was present in two patients. MRI showed ION hypertrophy and hyperintensity in five patients (unilateral, 4; bilateral, 1). Patients' demographic and clinical data are summarized in Table 1.

**Table 1:** Demographic and clinical data.

**Patient number**	**Age (years)**	**Gender**	**MRI**	Disease duration^*^(months)	**Neurological findings**	**Ocular motor findings**
1	66	F	Right pontine hemorrhage (C), right ION hypertrophy and hyperintensity	59	Dysarthria, dysphagia, left hemiparesis and hemiataxia, PT and associated facial movements	Pendular vertical and horizontal OD=OS nystagmus, bilateral INO
2	83	F	Right cerebellar hemorrhage (AVM), bilateral ION hypertrophy and hyperintensity	43	Dysarthria, generalized ataxia, PT and associated facial movements	Pendular vertical and torsional OD>OS nystagmus, jerk right beating nystagmus
3	59	F	Right pontine hemorrhage (C), right ION hypertrophy and hyperintensity	54	Dysarthria, left sensory loss, hemiparesis and hemiataxia, PT and associated facial movements	Pendular vertical OD>OS, torsional OD < OS, horizontal OD nystagmus
4	67	F	Right pontine hemorrhage (H), right ION hypertrophy and hyperintensity	78	Left hemiparesis and hemiataxia, PT	Pendular vertical and torsional OD < OS nystagmus, jerk left beating nystagmus, right one and half syndrome
5	77	M	Right pontine hemorrhage (C), no ION abnormality	36	Generalized ataxia, PT	Pendular vertical and torsional OD>OS nystagmus
6	59	F	Left pontine hemorrhage (C), left ION hypertrophy and hyperintensity	41	Right hemiparesis and hemitremor, PT	Pendular vertical and torsional OD>OS nystagmus, left one and half syndrome

*F, Female; M, Male; MRI, Magnetic Resonance Imaging; ION, Inferior Olivary Nucleus; C, Cavernoma; AVM, Arterio-Venous Malformation; H, Hypertension; PT, Palatal Tremor; INO, Internuclear Ophthalmoplegia; OD, Right eye; OS, Left eye*.

* *Time from hemorrhage to study entrance*.

### Ocular motor data

**Figure 1:**
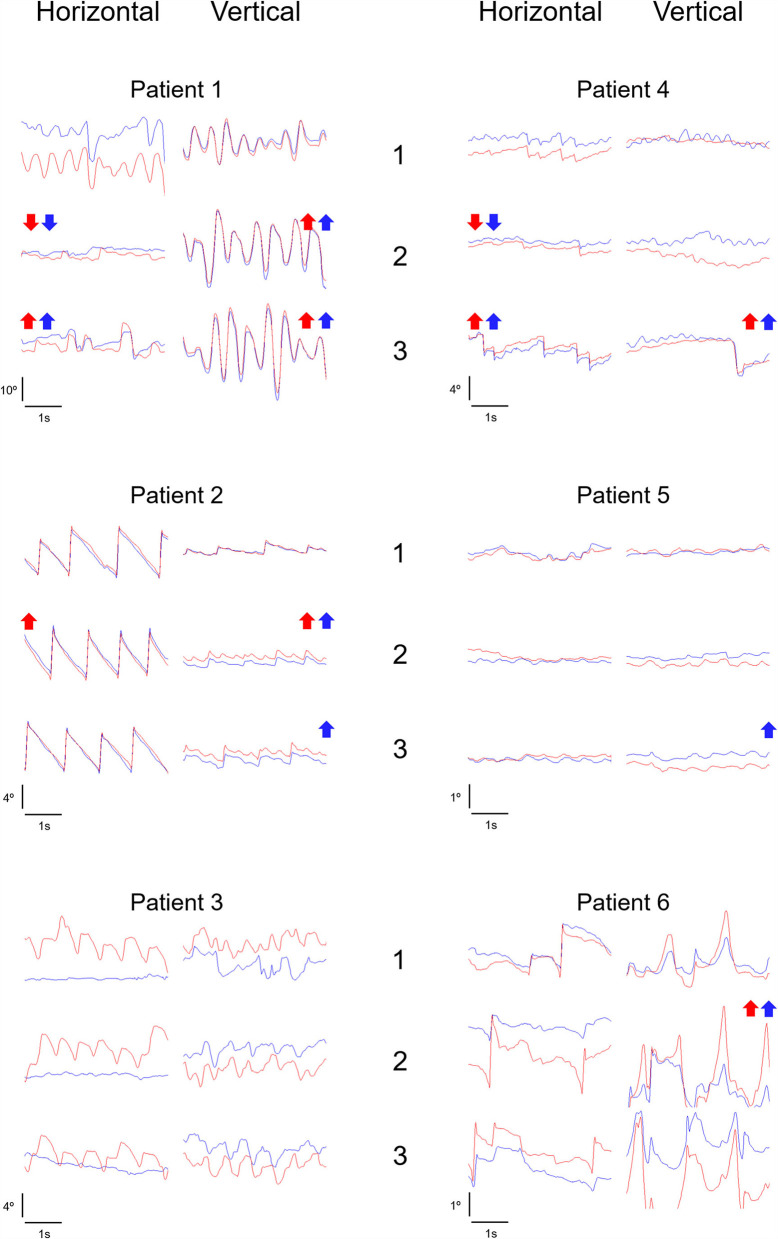
Ocular motor data. Nystagmus horizontal and vertical representative tracings in visits 1, 2, and 3 are depicted for each patient. Arrows up represent a >50% increase (i.e., worsening) in amplitude and/or velocity of nystagmus (red arrow, right eye; blue arrow, left eye), relative to the previous visit. Arrows down represent a >50% decrease (i.e., improvement) in amplitude and/or velocity of nystagmus (red arrow, right eye; blue arrow, left eye), relative to the previous visit. Red tracing, right eye. Blue tracing, left eye.

At baseline (visit 1), the mean vertical velocity and amplitude of nystagmus were 10.3–11.6°/s and 2.0–2.3°, mean horizontal velocity and amplitude of nystagmus were 9.4–13.8°/s and 2.8–3.7°, and mean frequency of nystagmus was 2.3 Hz. After 2 months of memantine intake (visit 2), for the horizontal plane, a 50% improvement of nystagmus velocity and/or amplitude in one or two eyes was seen in two patients, and a >50% worsening nystagmus velocity and/or amplitude in one or two eyes was seen in one patient. In contrast, for the vertical plane, 3 patients demonstrated a 50% worsening of nystagmus velocity and/or amplitude in one or two eyes, while the remaining patients showed no relevant change. After 6 months of memantine intake (visit 3), for the horizontal plane, two patients showed a 50% worsening of nystagmus velocity and/or amplitude in one or two eyes, while in the remaining patients, no relevant change was observed. For the vertical plane, four patients showed a 50% worsening of nystagmus velocity and/or amplitude in one or two eyes, while the remaining evidenced no relevant change. When performing group analysis, the mean vertical velocity and amplitude of the left eye significantly worsened from visit 2 to visit 3 (*p* = 0.028). Nystagmus frequency remained relatively unchanged throughout the study (*p* = 0.074). Ocular motor data is detailed in Figure 1, Table 2.

**Table 2:** Ocular motor data.

	**Plane**	**Eye**	**V1**	**V2**	**Change V1 vs. V2 (number of patients) (Worsening, improvement)**	**V3**	**Change V2 vs. V3 (number of patients) (Worsening, improvement)**	***P***	***P* (V1 vs. V2)**	***P* (V2 vs. V3)**	***P* (V1 vs. V3)**
Mean velocity+/-SD (Degrees per second)	Vertical	OS	10.3+/−14.7	14.8+/−24.6	(3,0)	23.1+/−42.7	(3,0)	***0.038***	*0.345*	***0.028***	*0.116*
		OD	11.6+/−17.7	15.0+/−22.0	(2,0)	23.7+/−43.8	(1,0)	*0.247*			
	Horizontal	OS	9.4+/−13.1	4.9+/−5.5	(0,2)	6.9+/−6.8	(2,0)	*0.438*			
		OD	13.8+/−18.2	7.4+/−6.9	(1,2)	8.9+/−8.1	(2,0)	*0.580*			
Mean amplitude+/-SD (Degrees)	Vertical	OS	2.0+/−3.0	3.1+/−5.8	(1,0)	5.4+/−10.7	(3,0)	***0.042***	*0.600*	***0.028***	*0.075*
		OD	2.3+/−3.7	3.2+/−5.3	(3,0)	4.4+/−8.0	(2,0)	*0.607*			
	Horizontal	OS	2.8+/−3.6	1.4+/−2.4	(0,1)	2.0+/−2.5	(2,0)	*0.337*			
		OD	3.7+/−4.1	2.3+/−2.2	(0,1)	5.0+/−7.2	(2,0)	*0.607*			
Frequency+/-SD (Hertz)	Frequency	OU	2.3+/−0.8	2.2+/−0.8	–	2.1+/−0.9	-	*0.074*			

*SD, Standard deviation; OS, Left eye; OD, Right eye; V1, Visit 1; V2, Visit 2; V3, Visit 3*.

*Significant differences are marked in bold*.

### Visual data

At baseline, mean BCVA was 0.4 and 0.3 in the right and left eye respectively. During the study, there was a significant improvement in BCVA in both eyes (*p* = 0.047, *p* = 0.017). In post analysis, this was most evident for the left eye, between visits 1 and 3 (*p* = 0.028), while a similar trend was observed for the right eye between visits 1 and 2, and 1 and 3. Throughout the study, SVV progression among patients was unpredictable, as reflected in group analysis (*p* = 0.513). Both SVV worsening, improvement, or no relevant change were observed. In two patients there was actually a relevant shift in SVV toward the opposite side (patients 2 and 3) (see Supplementary Table 1 for details). The mean NEI-VFQ-25 score at baseline was 63.8 and there was no significant change during the study (*p* = 0.223). Visual data is detailed in Table 3. Subjective improvement of oscillopsia was reported in three patients (patients 3, 4, and 6) and remained throughout the study.

**Table 3:** Visual data.

	**Eye**	**V1**	**V2**	**V3**	***P***	**P (V1 vs. V2)**	**P (V2 vs. V3)**	**P (V1 vs. V3)**
Mean visual acuity +/–SD (Decimal scale)	OD	0.4+/−0.1	0.6+/−0.2	0.6+/−0.3	***0.047***	*0.067*	*0.593*	*0.065*
	OS	0.3+/−0.1	0.5+/−0.2	0.6+/−0.3	***0.017***	*0.109*	*0.225*	***0.028***
Mean subjective visual vertical+/–SD (Degrees^*^)	OU	6.6+/−5.3	5.2+/−2.8	5.4+/−4.0	*0.513*			
Mean NEI–VFQ−25 score+/–SD		63.8+/−22.7	66.7+/−18.2	71.4+/−19.1	*0.223*			

*SD, Standard Deviation; OS, Left eye; OD, Right eye; NEI-VFQ-25, 25-Item National Eye Institute Visual Functioning Questionnaire; V1, Visit 1; V2, Visit 2; V3, Visit 3*.

* *Torsional degrees away from 0, regardless of the direction of deviation (i.e., clockwise or counter-clockwise)*.

*Significant differences are marked in bold*.

### Tremor data

Mean PT frequency ± SD remained unchanged during the study (Visit 1, 9.8 ± 2.7 Hz; visit 2, 10.3 ± 2.4 Hz; visit 3, 11.0 ± 3.8; *p* = 0.568). Three patients had PT propagation to facial muscles (patients 1, 2, and 3). At baseline, EMG-derived frequency was 2.5 Hz, 3.0 Hz, and 2.0 Hz, respectively, and did not significantly change between visits (data not shown).

### Correlations

At baseline, BCVA in both eyes negatively correlated with nystagmus frequency (*r* = −0.833, *p* = 0.039; *r* = −0.841, *p* = 0.036), BCVA in the left eye negatively correlated with a horizontal velocity of nystagmus in the left eye (*r* = −0.841, *p* = 0.036), and NEI-VFQ-25 score negatively correlated with vertical and horizontal velocity (*r* = −0.886, *p* = 0.019; *r* = −0.886, *p* = 0.019) and horizontal amplitude of nystagmus in the right eye (*r* = −0.829, *p* = 0.042). In visit 2, BCVA in the right eye negatively correlated with a horizontal velocity and amplitude of nystagmus in the right eye (*r* = −0.820, *p* = 0.046; *r* = −0.820, *p* = 0.046), BCVA in the left eye negatively correlated with a horizontal velocity and amplitude of nystagmus in the left eye (*r* = −0.899, *p* = 0.015; *r* = −0.812, *p* = 0.049), and NEI-VFQ-25 score negatively correlated with a horizontal velocity of nystagmus in the left eye (*r* = −0.886, *p* = 0.019) and positively correlated with BCVA in both eyes (*r* = 0.880, *p* = 0.021; *r* = 0.928, *p* = 0.008). Finally, at visit 3, BCVA in the right eye negatively correlated with a horizontal velocity of nystagmus in the right eye (*r* = −0.883, *p* = 0.020), BCVA in the left eye negatively correlated with a horizontal velocity and amplitude of nystagmus in the left eye (*r* = −0.928, *p* = 0.008; *r* = −0.899, *p* = 0.015), and NEI-VFQ-25 score negatively correlated with a horizontal and vertical velocity of nystagmus in both eyes (*r* = −0.943, *p* = 0.005; *r* = −0.829, *p* = 0.042; *r* = −0.829, *p* = 0.042; *r* = −0.829, *p* = 0.042) and a horizontal amplitude of nystagmus in the right eye (*r* = −0.886, *p* = 0.019). The 3 patients who reported subjective improvement of oscillopsia showed disparate outcomes in terms of BCVA, nystagmus parameters, and NEI-VFQ-25 score.

Memantine 20 mg per day was generally well tolerated, with only one patient reporting transient drowsiness at the beginning of the treatment.

## Discussion

We studied the effectiveness of memantine 20 mg per day for 6 months in OPT patients, focusing on the detection of putative distinctive effects on horizontal and vertical components of nystagmus, which could further help to explain the idiosyncratic subjective visual response previously seen in OPT patients. Importantly, we also aimed to ascertain if the effect of memantine on nystagmus and associated phenomena, including visual acuity, quality of life, estimation of gravity, and palatal/facial movements, was sustained over a period of 6 months.

Indeed, after 2 months, memantine improved the velocity and/or amplitude of nystagmus, but *only* along the horizontal plane, and just in two patients, while the vertical component worsened in four patients. At 6 months, improvement of nystagmus along the horizontal plane was not sustained in the two patients previously showing improvement, and there was an overall worsening of both horizontal and vertical amplitude and/or velocity of nystagmus at the group level. In contrast, visual acuity significantly improved throughout the study. Still, vision-targeted, health-related quality of life remained unchanged. Moreover, despite the apparent discrepancies in the long term between nystagmus intensity, visual acuity, and quality of life, the above parameters tended to correlate with each other at each visit. Oscillopsia improvement could not be consistently correlated with any of the above parameters. Finally, nystagmus frequency, gravity estimation, and palate tremor frequency did not seem to be influenced by memantine.

PN in OPT is thought to be due to a synchronized discharge of ION neurons. Further maladaptive learning by the cerebellar cortex is believed to amplify PN amplitude and frequency irregularity. The blockade of NMDA receptors by memantine seems to exert its major effect in the cerebellum, at the projections of the ION to the deep cerebellar nuclei, of the climbing fibers to the Purkinje neurons, and/or those of the parallel fibers onto the Purkinje neurons, ultimately leading to decreased Purkinje cells/cerebellar output (2, 13). This might explain why memantine in our and others' work was able to reduce PN amplitude and/or velocity but not its fundamental frequency (5–7). Specifically, PN amplitude seems to be a direct product of cerebellar output, while PN frequency is believed to be set by the intrinsic properties of the ION neurons the latter feature probably less amenable to memantine's effect (a similar conclusion may be drawn for the pathogenesis of palatal tremor frequency, which was also unaffected by memantine) (7). However, in our study, the improvement in nystagmus velocity and/or amplitude seemed to be distinct for the horizontal and vertical planes of nystagmus since the improvement was only seen along the horizontal plane and never along the vertical. Albeit rarely the focus of analysis in OPT-related nystagmus, there has been anecdotal data showing that responses to memantine might in fact be distinct between the horizontal, vertical, and torsional planes of PN. Specifically, the frequency irregularity of PN seems to improve in general with the use of memantine, but not along the horizontal plane (7). We hypothesize that memantine in our and others' cases might have exerted its action predominantly on projections from ION to the deep cerebellar nuclei carrying signals from independent olivary generators, giving rise exclusively to horizontal eye movements while showing less or no influence on projections signaling vertical and torsional eye movements (2). Whether such selective effect of memantine on fibers carrying signals for horizontal eye movements could be dosage-dependent, i.e., particularly seen when using lower dosages of memantine is highly speculative and lacks clinical and experimental evidence. Another non-mutually exclusive explanation for distinct/opposite responses to memantine depending on nystagmus plane is an increase in the nystagmus waveform randomness (i.e., nystagmus waveform shape from trial to trial) after memantine use, which might influence final/mean nystagmus velocity/amplitude waveform in different planes (8). Importantly, one cannot exclude that, particularly in patients with mild nystagmus, the aforementioned changes in nystagmus over time might reflect natural fluctuations. Still, if any improvement can be associated with the use of memantine, this was only seen for the horizontal plane, and it was not sustained, only being observed transiently.

While near visual acuity subjectively improved throughout the study, vision-related quality of life remained unchanged. This is in agreement with previous data (5). This finding either suggests that the amount of amplitude reduction of nystagmus was not adequate to provide a benefit in the visual quality of life, or other factors, besides visual acuity, might influence the overall visual function of OPT patients. Such factors might include memantine's “unwanted” effect on the PN waveform randomness (i.e., increase in waveform shape heterogeneity), coexisting ocular motor deficits also impairing vision (e.g., internuclear ophthalmoplegia, gaze palsies, vestibular deficits, etc.), and/or the presence of distinct/opposite effects of memantine on OPT-related nystagmus planes (i.e., horizontal, vertical and/or torsional) as shown in the present study (5, 7, 8). In addition, it must also be stressed that while NEI-VFQ-25 has been used before to evaluate OPT patients, this tool has not been specifically designed to assess the functional consequences of nystagmus (5). Still, the NEI-VFQ-25 global score correlated with visual acuity in one or both eyes at specific timepoints in our study, suggesting a weak, but nevertheless real association between both. Indeed, our results are in agreement with previous work showing improvement of visual acuity in OPT patients after memantine. Distance visual acuity has been previously shown to modestly improve in 6 out of 11 eyes after 2 weeks of memantine 40 g per day (6, 8). In another study using a similar protocol, only near visual acuity improved on memantine (5). Importantly, in our study, near visual acuity correlated with nystagmus parameters at specific timepoints. This finding emphasizes that nystagmus intensity by itself seems to directly impair vision in OPT (5). Other factors which might also be influencing visual acuity outcomes in the current work include improvement of torsional velocity/amplitude of nystagmus (which was not quantitatively measured), and patient's level of cooperation and/or learning effects throughout the study. Oscillopsia improved in half of our patients. Previously, oscillopsia was either unchanged or modestly improved in 4 OPT patients (6). Importantly, in another study, this effect was only observed for distance oscillopsia (5). As we did not perform a separate assessment for distance and near oscillopsia, further interpretation of our results is precluded.

Taking into account the side effects/tolerability profile of memantine 40 mg per day, the dosage of 20 mg per day was chosen for the current study (6). Indeed, memantine 40 mg per day has been discontinued in 18.8% of patients in one study, reduced to 20 mg per day in one patient in another study, and voluntarily stopped or reduced by all OPT patients once the above studies ended (5, 6). Rather expectedly, using half of the dosage, only one patient reported mild imbalance in our study, which was nevertheless tolerated. The use of a smaller dosage could eventually explain the lack of benefit in nystagmus parameters in some of our patients, but the memantine-related improvement on OPT-related nystagmus has nevertheless been demonstrated when using the dosage of 20 mg per day for 2 weeks (6). Still, our results are difficult to compare with those from previous studies, due to our particularly long study duration, i.e., 6 months, vs. <1 month in previous works (5–7). This is actually one of the main strengths of our study. Such a long duration allowed us to demonstrate that any potential benefit on nystagmus intensity seen early on in the study was not relevantly sustained over time, thus providing important data concerning the long-term management of OPT patients.

Finally, gravity perception has been seldomly investigated in OPT patients (4). In our work, SVV deviations were pathological in five patients and further evidenced significant shifts over time in two patients. The above findings seem to reflect the simultaneous contribution of co-existent lesions affecting the vestibular graviceptive pathways and an over-excitation of the dentate nucleus due to a lack of ION inhibition (4). Not surprisingly, memantine showed no relevant effect on SVV, since none of the aforementioned mechanisms seem to be strongly dependent on NMDA receptor-related modulation (4).

There are several limitations to the current study. Apart from the limited size of the sample reflecting the rarity of OPT, further analyses on nystagmus frequency irregularity, conjugacy, waveform randomness, and assessment of distance visual acuity were not performed and could have provided further insight. Similarly, while the torsional component of nystagmus did not seem to relevantly change before and after treatment in any patient (data not shown), quantitative analysis of the torsional plane of nystagmus was not performed. Additionally, the lack of a placebo treatment design in the current study did not allow us to control and evaluate the magnitude of a potential placebo effect and/or normal nystagmus fluctuation over time in our results (14). This issue becomes particularly relevant in patients whose nystagmus was mild, where minor changes might naturally occur over time.

We conclude that the use of memantine 20 mg per day for 6 months in our OPT patients showed a modest improvement in nystagmus horizontal amplitude and velocity, which was not sustained over time. Although there was a sustained improvement in visual acuity, vision-related quality of life remained unchanged. A long-term (e.g., 6 months) placebo-controlled trial using different dosages of memantine (e.g., 20 and 40 mg), including analyses of nystagmus in each plane and waveform randomness, and assessments of near and distance visual acuity, near and distance oscillopsia, and quality of life as main outcomes, in a larger sample of patients, is needed to further elucidate the role of memantine in OPT.

## Data availability statement

The raw data supporting the conclusions of this article will be made available by the authors, without undue reservation.

## Ethics statement

The studies involving human participants were reviewed and approved by Comissão de Ética para a Saúde. The patients/participants provided their written informed consent to participate in this study. Written informed consent was obtained from the individual(s) for the publication of any potentially identifiable images or data included in this article.

## Author contributions

AIM and RS contributed to the acquisition, analysis and interpretation of the data, and drafting of the manuscript. AJ, CD, CF, and JS-F contributed to the acquisition, analysis, and interpretation of the data. DP, LN, and AM contributed to the acquisition, analysis, interpretation of the data, and study concept and design. JL contributed to the acquisition, analysis, interpretation of the data, study supervision, concept, design, and critical revision of manuscript for intellectual content. All authors contributed to the article and approved the submitted version.

## Conflict of interest

The authors declare that the research was conducted in the absence of any commercial or financial relationships that could be construed as a potential conflict of interest.

## Publisher's note

All claims expressed in this article are solely those of the authors and do not necessarily represent those of their affiliated organizations, or those of the publisher, the editors and the reviewers. Any product that may be evaluated in this article, or claim that may be made by its manufacturer, is not guaranteed or endorsed by the publisher.

## References

[1] Tilikete C Desestret V. Hypertrophic olivary degeneration and palatal or oculopalatal tremor. Front Neurol. (2017) 8:302. 10.3389/fneur.2017.00302 28706504 PMC5490180

[2] Shaikh AG Hong S Liao K Tian J Solomon D Zee DS. Oculopalatal tremor explained by a model of inferior olivary hypertrophy and cerebellar plasticity. Brain. (2010) 133:923–40. 10.1093/brain/awp323 20080879 PMC2842510

[3] Goyal M Versnick E Tuite P Cyr JS Kucharczyk W Montanera W. Hypertrophic olivary degeneration: metaanalysis of the temporal evolution of MR findings. Am J Neuroradiol. (2000). 21:1073–7. 10871017 PMC7973904

[4] Tarnutzer AA Palla A Marti S Schuknecht B Straumann D. Hypertrophy of the inferior olivary nucleus impacts perception of gravity. Front Neurol. (2012). 3:79. 10.3389/fneur.2012.00079 22593754 PMC3350027

[5] Nerrant E Abouaf L Pollet-Villard F Vie A Vukusic S Berthiller J. Gabapentin and memantine for treatment of acquired pendular nystagmus: effects on visual outcomes. J Neuroophthalmol. (2020) 40:198–206. 10.1097/WNO.0000000000000807 31169568

[6] Thurtell MJ Joshi AC Leone AC Tomsak RL Kosmorsky GS Stahl JS. Crossover trial of gabapentin and memantine as treatment for acquired nystagmus. Ann Neurol. (2010) 67:676–80. 10.1002/ana.21991 20437565 PMC3064518

[7] Shaikh AG Thurtell MJ Optican LM Leigh RJ. Pharmacological tests of hypotheses for acquired pendular nystagmus. (2011) 1233:320–6. 10.1111/j.1749-6632.2011.06118.x 21951011 PMC3187918

[8] Theeranaew W Thurtell MJ Loparo K Shaikh AG. Gabapentin and memantine increases randomness of oscillatory waveform in ocular palatal tremor. J Comput Neurosci. (2020) 49:319–31. 10.1007/s10827-020-00753-6 32621105

[9] Zwergal A Rettinger N Frenzel C Dieterich M Brandt T Strupp M. A bucket of static vestibular function. Neurology. (2009) 72:1689–92. 10.1212/WNL.0b013e3181a55ecf 19433743

[10] Das A Quartilho A Xing W Bunce C Rubin G MacKenzie K. Visual functioning in adults with Idiopathic Infantile Nystagmus Syndrome (IINS). Strabismus. (2018) 26:203–9. 10.1080/09273972.2018.1526958 30325248

[11] Kelly JP Phillips JO Weiss AH. Does eye velocity due to infantile nystagmus deprive visual acuity development? J AAPOS. (2018) 22:50–55.e1. 10.1016/j.jaapos.2017.10.008 29288837

[12] Kori AA Robin NH Jacobs JB Erchul DM Zaidat OO Remler BF. Pendular nystagmus in patients with peroxisomal assembly disorder. Arch Neurol. (1998) 55:554–8. 10.1001/archneur.55.4.554 9561985

[13] Strupp M Thurtell MJ Shaikh AG Brandt T Zee DS Leigh RJ. Pharmacotherapy of vestibular and ocular motor disorders, including nystagmus. J Neurol. (2011) 258:1207–22. 10.1007/s00415-011-5999-8 21461686 PMC3132281

[14] Theeranaew W Kim HJ Loparo K Kim JS Shaikh AG. Hyperventilation Increases the Randomness of Ocular Palatal Tremor Waveforms. Cerebellum. (2021) 20:780–7. 10.1007/s12311-020-01171-1 32737797

