# Supplementary material for: A 6-month trial of memantine for nystagmus and associated phenomena in oculopalatal tremor
Source: Front Neurol. 2022 Aug 17;13:921341. doi: 10.3389/fneur.2022.921341 (PMC9428258; doi:10.3389/fneur.2022.921341)
Supplement: Supplementary file 1 [file Table_1.DOCX]

Supplemental Table 1. Subjective visual vertical.

| Patient number | V1 | V2 | V3 |
| --- | --- | --- | --- |
| 1 | -10.25 | -9.00 | -3.25 |
| 2 | -2.08 | 5.17 | 10.42 |
| 3 | -14.67 | -5.75 | 10.17 |
| 4 | -8.25 | -7.33 | -5.08 |
| 5 | -4.67 | -3.75 | -2.90 |
| 6 | -0.25 | -0.75 | -0.70 |

Negative values represent counter-clockwise deviation (patient’s perspective) of subjective visual vertical (SVV) while positive values represent clockwise deviation (patient’s perspective) of SVV
